# Differential regulation of iron chelator-induced IL-8 synthesis via MAP kinase and NF-κB in immortalized and malignant oral keratinocytes

**DOI:** 10.1186/1471-2407-7-176

**Published:** 2007-09-13

**Authors:** Hwa-Jeong Lee, Jun Lee, Sun-Kyung Lee, Suk-Keun Lee, Eun-Cheol Kim

**Affiliations:** 1 Department of Oral & Maxillofacial Pathology, College of Dentistry, Wonkwang University, Iksan, Republic of Korea; 2 Department of Oral and Maxillofacial Surgery, College of Dentistry, Wonkwang University, Iksan, Republic of Korea; 3 Department of Oral Pathology, College of Dentistry, Kangnung National University, Gangneung, Republic of Korea

## Abstract

**Background:**

Interleukin-8 (IL-8) is a cytokine that plays an important role in tumor progression in a variety of cancer types; however, its regulation is not well understood in oral cancer cells. In the present study, we examined the expression and mechanism of IL-8 in which it is involved by treating immortalized (IHOK) and malignant human oral keratinocytes (HN12) cells with deferoxamine (DFO).

**Methods:**

IL-8 production was measured by an enzyme-linked immunoabsorbent assay and reverse transcriptase-polymerase chain reaction (RT-PCR) analysis. Electrophoretic mobility shift assays was used to determine NF-κB binding activity. Phosphorylation and degradation of the I-κB were analyized by Western blot.

**Results:**

IHOK cells incubated with DFO showed increased expression of IL-8 mRNA, as well as higher release of the IL-8 protein. The up-regulation of DFO-induced IL-8 expression was higher in IHOK cells than in HN12 cells and was concentration-dependent. DFO acted additively with IL-1β to strongly up-regulate IL-8 in IHOK cells but not in HN12 cells. Accordingly, selective p38 and ERK1/2 inhibitors for both kinases abolished DFO-induced IL-8 expression in both IHOK and HN12 cells. Furthermore, DFO induced the degradation and phosphorylation of IκB, and activation of NF-κB. The IL-8 inducing effects of DFO were mediated by a nitric oxide donor (S-nitrosoglutathione), and by pyrrolidine dithiocarbamate, an inhibitor of NF-κB, as well as by wortmannin, which inhibits the phosphatidylinositol 3-kinase-dependent activation of NAD(P)H oxidase.

**Conclusion:**

This results demonstrate that DFO-induced IL-8 acts via multiple signaling pathways in immortalized and malignant oral keratinocytes, and that the control of IL-8 may be an important target for immunotheraphy against human oral premalignant lesions.

## Background

Approximately 40,000 cases of cancer of the mouth and pharynx are reported annually in the United States, and it is the sixth most common cancer type worldwide. Over 90% of oral-pharyngeal cancers are squamous cell carcinomas (SCC). The 5-year survival rate for this type of cancer is approximately 50% and has not markedly improved in the past 30 years [1,2]. Several chemotherapy regimes have been clinically applied to treat oral-pharyngeal cancers, but none have shown to significantly improve prognoses [3,4]. Thus, a safe and effective anticancer target is needed to treat oral cancer.

Iron is a critical nutritional element that is essential for a variety of important biological processes including cell growth and differentiation, electron transfer reactions, and oxygen transport, activation, and detoxification [5]. Iron also has a major effect on neoplastic cell growth due to its catalytic effect on the formation of hydroxyl radicals, its suppression of the activity of host defense cells, and its role in the promotion of cancer cell multiplication [6,7]. Indeed, iron chelation by deferoxamine (DFO), a bacterial siderophore, has been shown to inhibit the growth of and/or to induce apoptosis in malignant leukemia, neuroblastoma, melanoma, hepatoma, Kaposi's sarcoma, and cervical cancer cell lines [8-14].

*In vitro *human cell lines as well as oral SCC tumors have been used to demonstrate the increased level of some pro-inflammatory, pro-angiogenic NF-κB dependent cytokines in oral-pharyngeal cancers. These include TNF-α, IL-1, IL-6, IL-8, GM-CSF, and VEGF, which have been shown to be highly elevated in the local milieu of SCC [15-18]. Evidence exists that the production of these cytokines is unregulated in oral-pharyngeal SCC and that they have roles in cell growth, invasion, interruption of tumor suppression, immune status, and survival [15,16]. It is unknown, however, whether these mediators are critical for the development and/or growth of tumors, and whether they create a permissive environment for the progression of malignancies [17-19].

Interleukin-8 (IL-8) was originally identified as a neutrophil chemotatic factor in the supernatants of activated human monocytes [20,21]. IL-8 is a pluripotent pro-tumorigenic cytokine that is known to induce angiogenesis, tumor cell proliferation, and tumor cell migration [22-25]. The local expression of IL-8 in the tumor environment likely plays an important role in cancer progression and metastasis [26]. Therefore, it is essential to define the activation pathways in which iron chelation controls IL-8 expression in oral cancer cells in order to develop appropriate therapeutic regimes.

We recently discovered that DFO inhibited the proliferation and induced the apoptosis of immortalized human oral keratinocytes (IHOK) and oral cancer cells [27] We also reported that p38 and ERK MAP kinase mediated DFO-induced apoptosis and the suppression of differentiation in IHOK and oral cancer cells [28]; however, an immunomodulatory role of IL-8 against the iron chelator DFO has not been reported in oral cancer and IHOK cells. The purpose of this study was to determine whether DFO affects IL-8 signaling and stabilization through mitogen-activated protein (MAP) kinase, NF-κB pathways, posttranscriptional mechanisms, and multiple intracellular signal transduction in immortalized and malignant oral keratinocytes.

## Methods

### Materials

DFO (deferoxamine), mimosine (MIM), ferric citrate (FC), alkaline-phosphatase-conjugated monoclonal mouse anti-rabbit IgG, and *p*-nitrophenyl phosphate tablets were purchased from Sigma-Aldrich (St. Louis, MO). All reagents and media for tissue culture experiments were tested for their LPS contents with a colorimetric *Limulus *amebocyte lysate assay (detection limit 10 pg/Mℓ Sigma-Aldrich). Human IL-1β was obtained from Invitrogen (Carlsbad, CA). TNF-α and polyclonal goat anti-human IL-8 were obtained from R & D Systems (Minneapolis, MN). SB203580 and PD98059 were purchased from Calbiochem (La Jolla, CA). Polyclonal rabbit anti-human IL-8 was from Endogen (Woburn, MA). Abs against HRP-conjugated anti-rabbit IgG was from Amersham Biosciences (Little Chalfont, U.K.). Anti-human I-κBα or antiphospho-I-κBα was from Santa Cruz Biotechnology (Santa Cruz, CA). Dulbecco's modified Eagle's medium (DMEM), fetal bovine serum (FBS), and other tissue culture reagents were purchased from Gibco BRL (Grand Island, NY).

### Cell culture

Informed consent was obtained from all subjects. The study protocol was approved by ethical committee and institutional review boards of Wonkwang University, dental college, Iksan, Republic of Korea (WKDIRB 20048-13). This study complies with the Helsinki Declaration in its recent Korean version. The trial will also be carried out in keeping with local legal and regulatory requirements.

HPV-immortalized human oral keratinocytes (IHOK) were in vitro-established cell lines, which immortalized by transfection of normal human oral keratinocytes with PLXSN vector containing the E6/E7 open reading frames of HPV type 16 as previously described [27-29]. The IHOK cells were obtained from Dr. Myung-Hee Park (NIDCR, NIH, MD), and cultured in the keratinocyte growth medium (KGM, Gibco, Grand Islands, NY) supplemented with 2 mL of bovine pituitary extract (13 mg/ml), 0.5 ml each of hydrocortisone (0.5 mg/ml), human epidermal growth factor (0.5 μg/ml), insulin (5 mg/ml), epinephrine (0.5 mg/ml), transferrin (10 mg/ml), triiodothyronine (6.5 μg/ml), and GA-1000 and 0.05 mM CaCl_2_.

HN12 (= HNSCC12) cells were cultured in Dulbecco's modified Eagle's medium (DMEM, Biofluid, Rockville, MD) containing 10% fetal bovine serum (FBS, Gibco, USA) with 100 U/ml penicillin and 100 U/ml streptomycin (Life Technologies, Gaithersburg, MD). HN12 from metastatic carcinoma of the oral cavity [30] were obtained from the laboratory of Dr. John F. Ensley (Wayne State University). All the cell lines were grown at 37°C in a humidified atmosphere of 5% CO_2 _and 95% air. Cells were dissociated with 0.25% trypsin just before transfer for experiments and were counted using a hemocytometer.

### IL-8 measurement

IHOK and HN12 cells were seeded at 2 × 10^4 ^into 12-well plates (Nalge Nunc International, Rochester, NY) and cultured in serum-containing medium for 24 h prior to treatment. Cells were treated with fresh medium containing stimuli as indicated. The supernatants were collected, cleared by centrifugation, and kept at -20°C until evaluation by ELISA. For measurement of IL-8 concentrations in cell culture supernatants, 96-well microtiter plates (MaxiSorp; Nunc) were coated with 0.2 μg/well goat anti-human IL-8 Abs (R & D Systems) in 50 μℓ of PBS at 4°C overnight. All further steps were conducted at room temperature. After washing three times with PBS, nonspecific binding sites were blocked by incubation with 150 μℓ PBS + 1% BSA/0.05% Tween-20/well for 2 h. After three washes with PBS, 50 μℓ of samples or IL-8 standards were added and incubated for 2 h. As a second antibody, 0.05 μg/well polyclonal rabbit anti-human IL-8 (Endogen) was added and incubated for 2 h. As a third antibody, alkaline phosphatase-labeled monoclonal mouse anti-rabbit IgG (Sigma-Aldrich) was diluted in 50 μℓ of PBS + 0.1% BSA/0.05% Tween-20 to 1:50,000 and incubated for 2 h. Finally, alkaline phosphatase substrate *p*-nitrophenyl phosphate (Sigma-Aldrich) was added at a concentration of 1 mg/ml in 0.1 M glycine buffer (pH 10.4) containing 1 mM MgCl_2 _and 1 mM ZnCl_2_. After overnight incubation, plates were read at 405 nm on a microplate reader (Molecular Devices, Sunnyvale, CA). The detection limit of the ELISA was 30 pg/ml.

### RNA isolation and RT-PCR

Cells (5 × 10^5^) were grown in 60-mm culture dishes for 24 h prior to treatment, and incubated for 4–24 h in a fresh medium containing stimuli as indicated. After discarding growth medium, total RNA was isolated from cells using easy-Blue (iNtRON Biotechnology, Daejon, Korea), following the manufacturer's instructions. Reverse transcription of the RNA was performed using AccuPower RT PreMix (Bioneer, Daejon, Korea). One microgram of RNA and 20 pmol primers were preincubated at 70 degrees for 5 min and transferred to a mixture tube. The reaction volume was 20 μℓ. cDNA synthesis was performed at 42 degrees for 60 min, followed by RT inactivation at 94 degrees for 5 min. Thereafter, the RT-generated DNA (2–5μℓ) was amplified using AccuPower PCR PreMix (Bioneer). The primers used for cDNA amplification and PCR conditions were as follows: IL-8 [31], 5-ATGACTTCCAAGCTGGCCGTGGCT-3 (sense) and 5-TCTCAGCCCTCTTCAAAAACTTCTC-3 (antisense); GAPDH, 5-CG GAGTCAACGGATTTGGTCGTAT-3 (sense) and 5-AGCCTTCTCCA TGGTGGTGAAGAC-3 (antisense); an initial denaturation at 94 degrees for 5 min; 25 cycles being conducted for the time course measurements of IL-8 and GAPDH and 30 cycles for the detection of mRNA decay, each cycle with 30 s of denaturation at 94 degrees, 30 s of annealing at 62 degrees and 30 s of extension at 72 degrees; and a final dwell at 72 degrees for 7 min. The expected PCR products were 289 bp (for IL-8) and 306 bp (for GAPDH). PCR products were resolved on a 1.5% agarose gel and stained with ethidium bromide.

### Electrophoretic mobility shift assays

Cells (1 × 10^6^) were grown in 100-mm culture dishes and incubated for various times with DFO. Cells were mechanically scraped in PBS and washed, then 1 × 10^7 ^cells were resuspended in 500 μl of lysis buffer A (15 mM KCl, 10 mM HEPES, 2 mM MgCl_2_, 0.1 mM EDTA, 1 mM PMSF, 1 mM dithiothreitol (DTT), 10 μg/ml aprotinin, 2 μg/ml leupeptin, 0.1% NP-40, pH 7.6). Cell suspensions were then incubated for 10–15 min on ice with occasional vortexing, and centrifuged for 30 s to pellet nuclei, which were rinsed with wash buffer B (2 mM KCl, 25 mM HEPES, 0.1 mM EDTA, 1 mM PMSF, 1 mM DTT, 10 μg/ml aprotinin, 2 μg/ml leupeptin, pH 7.6) and incubated at 4°C for 20 min. Nuclear extracts were then prepared by centrifugation at 20 000 × *g *for 15 min in buffer C (25 mM HEPES, 0.1 mM EDTA, 20% glycerol, pH 7.6) and stored at -80° until used for EMSA. The probes contained the NF-kB or the AP-1 oligonucleotide consensus sequence and were labeled with γ-^32^P (Amersham Life Science) (3000 Ci/mmol, 250 μCi) using T4 polynucleotide kinase (Boehringer Mannheim). In competition assays, 100 × cold NF-kB competitor was added. The DNA-protein complex was separated on a non-denaturating 4% polyacrylamide gel in TBE buffer (Tris-HCl, boric acid, EDTA 2 mM, pH 8.0). After electrophoresis, the gel was dried and autoradiographed by overnight exposure to X-ray film.

### Cell extract preparation and Western blot analysis

IHOK and HN 12 cells (1 × 10^6^) were grown in 100 mm-dishes and incubated for 0.5–16 h in fresh medium containg stimuli as indicated. For the analysis of phosphorylation and degradation of the I-κB, stimulated cells were rinsed twice with ice-cold PBS and then lysed in ice-cold lysis buffer (50 mM Tris-HCl (pH 7.4), containing 150 mM NaCl, 1 % Nonidet P-40, 0.1 % SDS, 0.1 % deoxycholate, 5 mM sodium fluoride, 1 mM sodium orthovanadate, 1 mM 4-nitrophenyl phosphate, 10 μg/Mℓ leupeptin, 10 μg/Mℓ pepstatin A, and 1 mM 4-(2-aminoethyl)benzenesulfonyl fluoride). Cell lysates were centrifuged at 15,000 rpm for 20 min at 4°C, and the supernatant was mixed with a one-fourth volume of 4 × SDS sample buffer, boiled for 5 min, and then separated through a 12 % SDS-PAGE gel. After electrophoresis, proteins were transferred to a nylon membrane by means of Trans-Blot SD semidry transfer cell (Bio-Rad, Hercules, CA). The membrane was blocked in 5 % skim milk (1 h), rinsed, and incubated with primary antibody (for phosphorylated MAPKs or I-κB) in TBST and 3 % skim milk overnight at 4°C. Excess primary antibody was then removed by washing the membrane four times in TBST, and the membrane was incubated with 0.1 μg/Mℓ peroxidase-labeled secondary antibody (against rabbit) for 1 h. Following three washes in TBST, bands were visualized by ECL Western blotting detection reagents and exposed to x-ray film.

### Statistical analysis

Differences among groups were analyzed using one-way analysis of variance combined with the Bonferroni test. All values were expressed as means ± standard deviations, and differences were considered significant at *p *< 0.05.

## Results

### Effects of Iron chelators on IL-8 expression in IHOK and HN12 cells

**Figure 1:**
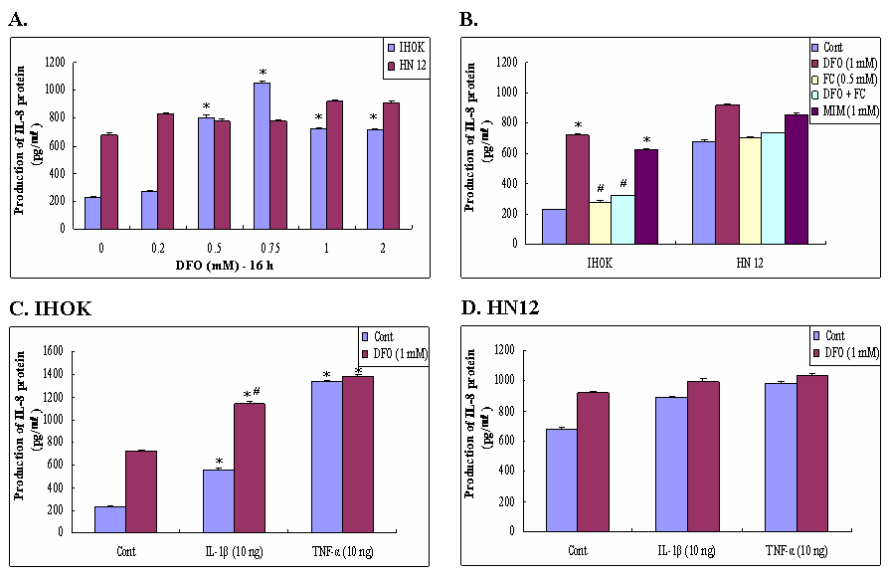
Effects of Iron chelator on IL-8 production in immortalized (IHOK) and malignant human oral keratinocytes (HN12). Cells were treated for 16 h with the indicated concentrations of DFO (0.2–2 mM) in IHOK and HN12 cells (A), or DFO (1.0 mM), FC (0.5 mM), MIM (1.0 mM) in IHOK and HN12 cells (B), IL-1 β (10 ng/ml), TNF-α (10 ng/ml), or combinations thereof in IHOK (C) and HN12 (D) cells. Levels of IL-8 secretion were determined by ELISA. Results are expressed as means ± SD of three independent experiments. Numbers below the gels represent the intensity of IL-8 mRNA relative to GAPDH mRNA.*: Statistically significant difference compared to control group, p < 0.05. #: Statistically significant difference compared to DFO group, *p *< 0.05.

We examined whether chelation of iron from immortalized human oral keratinocyte (IHOK) and oral squamous cell carcinoma cells (HN12) was sufficient to induce a signal that would increase the production IL-8. We found that IL-8 secretion was elevated upon exposure to DFO in immortalized IHOK cells, but no significant change in IL-8 concentration was seen in HN12 cells (Fig. 1A). The effect of DFO was concentration-dependent in the range of 0 to 2 mM; however, higher concentrations of DFO did not increase the production of IL-8. Maximal IL-8 production was achieved using 0.75 mM DFO in IHOK cells (Fig. 1A).

MIM is an iron chelator that is structurally distinct from DFO. MIM also induced IL-8 secretion in IHOK cells, but again, no significant change was observed in HN12 cells (Fig. 1B). Conversely, the addition of FC (Fe^3+^, 0.5 mM) significantly prevented DFO-induced IL-8 production (Fig. 1B), indicating that the target of DFO is specific for intracellular iron in IHOK cells. As shown in Fig. 1C, the IL-8 concentrations induced by DFO were comparable to those induced by IL-1β (10 ng/ml) and TNF-α (10 ng/ml), and combining DFO with IL-1β had an additive effect on IL-8 secretion in IHOK cells. Conversely, IL-8 secretion did not differ significantly between the DFO and the DFO plus cytokine treatments in HN12 cells (Fig. 1D).

**Figure 2:**
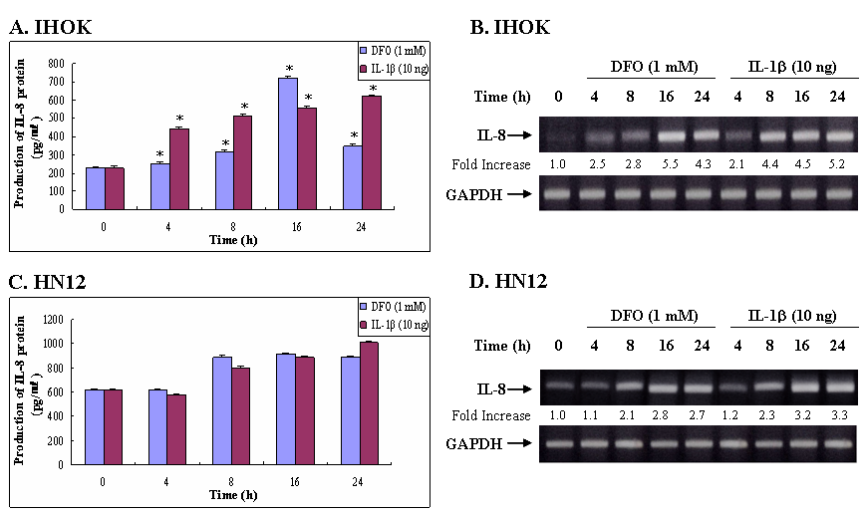
Iron chelator induces IL-8 protein secretion (A, C) and IL-8 mRNA accumulation (B, D) in IHOK and HN12 cells in a time-dependent manner. Cells were incubated with DFO (1.0 mM) or IL1-β (10 ng/ml) for the indicated time periods. Levels of IL-8 protein and mRNA were determined by ELISA and semiquantitative RT-PCR, respectively. Numbers below the gels represent the intensity of IL-8 mRNA relative to GAPDH mRNA. These data are representative of three independent experiments.

We found that DFO induced IL-8 secretion in IHOK and HN12 cells in a time-dependent manner (Fig. 2), and that maximal induction occurred after 16 h of incubation (Fig. 2A and 2C). This is different from that observed in IL-1β elicited IL-8 secretion, where a plateau of IL-8 reached within 24 h after IL-1β treatment in IHOK cells. We also found that the increase of IL-8 protein secretion appears to correspond to increased IL-8 mRNA levels in IHOK cells (Fig. 2B).

### Iron chelator induces IL-8 secretion in IHOK and HN12 cells via an NF-κB-dependent mechanism

**Figure 3:**
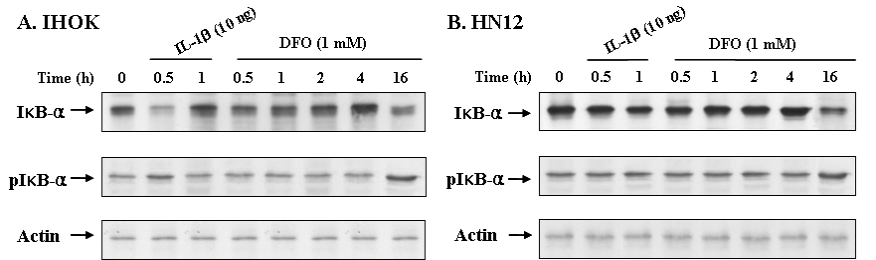
Iron chelator induced phosphorylated IκB-α in IHOK and HN12 cells on time dependent. Cells were treated with DFO (1.0 mM) or IL-1α (10 ng/ml) for the indicated time periods. Levels of IκB-α, p IκB-α were determined by Western blotting. The protein fraction was extracted, electrophoresed, transferred to membrane and blotted with respective antibodies. These data are representative of three independent experiments.

**Figure 4:**
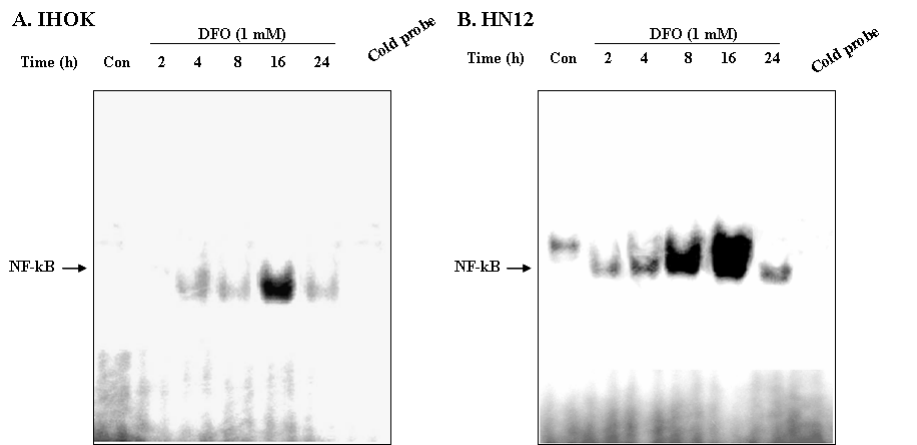
DFO induced the DNA binding activities of NF-kB in IHOK and HN12 cells. The nuclear extracts from the cells treated with DFO 1.0 mM at the indicated times were incubated with [γ-32P] ATP-labeled NF-kB probe, and analyzed by EMSA. The specificity of the bands was verified by adding a 10-fold excess of a completing unlabeled NF-kB probe (cold probe) to the 4 h DFO treated unclear proteins. The results were confirmed by two independent experiments.

The activation of NF-κB is usually associated with the phosphorylation of IκB-α. NF-κB is then released and translocated to the nucleus to activate targeted gene expression and the phosphorylated IκB-α is degraded by a proteasome. We examined the effect of DFO on NF-κB activation by measuring I-κBα and pI-κBα degradation (Fig. 3), and DNA binding of NF-κB (Fig. 4). To determine the levels of I-κBα and pI-κBα, IHOK and HN12 cells were incubated with 1.0 mM DFO for 0.5 to 16 h. Cell lysates were prepared to analyze the degradation and phosphorylation of I-κBα by Western blot analysis using anti-I-κBα and pI-κBα antibodies. As shown in Fig. 3, IL-1α treatment induced significant I-κBα degradation in 1 h whereas significant DFO-induced I-κBα degradation was only seen after 16 h in IHOK and HN12 cells.

Given that NF-κB activity is dependent on a steady concentration of I-κB, we investigated the effect of DFO on I-κB phosphorylation. Phosphorylated I-κB was detected 16 h after DFO stimulation in IHOK and HN12 cells (Fig. 3A and 3B). This demonstrated that the dissociation of NF-κB from I-κB plays a crucial role in NF-κB signal transduction, and that NF-κB was translocated to the nucleus when DFO was added to IHOK and HN12 cells.

### Iron chelator induces the DNA-binding activity of NF-κB in IHOK and HN12 cells

It has been demonstrated that NF-κB is the central regulator of IL-8 gene expression (32). We therefore examined the effect of DFO on the activation of NF-κB using the electrophoretic mobility shift assay (EMSA) to analyze the NF-κB binding activity of extracted proteins. The nuclear extracts were isolated from DFO-treated IHOK and HN12 cells, and NF-κB oligomers were used as probes. We observed that DFO-treated IHOK and HN12 cells showed increased DNA binding of NF-κB (Fig. 4A), and that maximum binding activity was detected after 16 h of incubation (Fig. 4B). These results indicate that an NF-κB dependent pathway regulates iron chelator-mediated IL-8 production in IHOK and HN12 cells.

### Both p38 and ERK1/2 activation contribute to iron chelator induced IL-8 production in IHOK and HN12 cells

**Figure 5:**
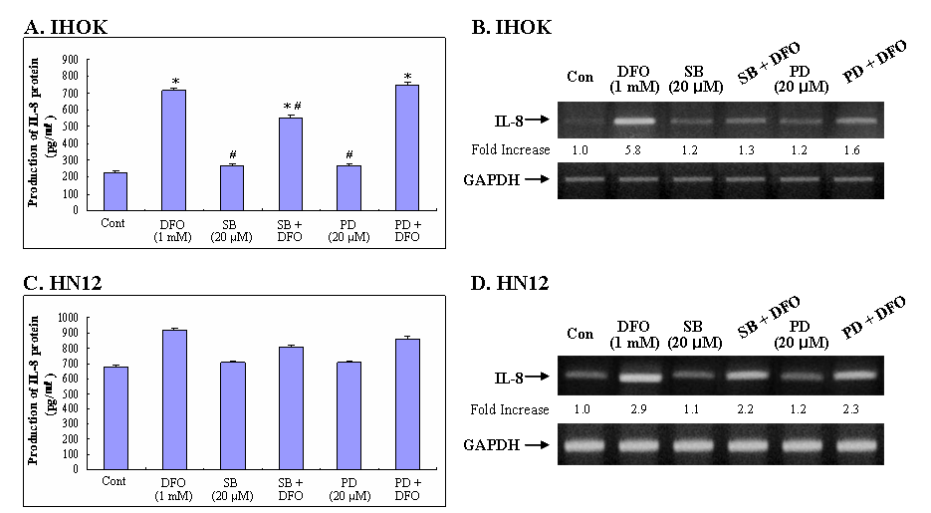
The effect of inhibitor for p38 and ERK MAPK on iron chelator induced IL-8 protein secretion and IL-8 mRNA in IHOK and HN12 cells. IHOK (A) & HN12 cells (C) were pretreated with ERK inhibitor PD98059 and the p38 inhibitor SB203580 for 1 h and followed by the treatment with DFO (1.0 mM) for 16 h. Levels of IL-8 secretion and IL-8 mRNA were determined by ELISA and RT-PCR in IHOK (B) & HN12 cells (D). Same procedure as described in the legend to Fig. 1 was performed. Results are expressed as means ± SD of three independent experiments. *: Statistically significant difference compared to control group: p < 0.05, #: Statistically significant difference compared to DFO group, *p *< 0.05.

Previous studies have shown that three MAP kinase subfamilies contribute to cell survival and the induction of apoptosis in some cell systems [33-36]. Furthermore, the activation of p38 and ERK MAP kinase was observed during iron deprivation-induced apoptosis in immortalized and malignant oral keratinocytes [28]. To examine whether the activation of p38 and ERK1/2 by DFO treatment contributes to the stimulatory effect of DFO on IL-8 production, we investigated the effects of pharmacological agents that modulate MAP kinase activities. IHOK and HN12 cells were treated for 48 h with DFO (1.0 mM), PD98059 (20 μM), SB203580 (20 μM), or combinations thereof (Fig. 5). Treatment of IHOK cells with SB203580, the selective inhibitor for p38, blocked DFO-induced IL-8 production whereas the ERK pathway inhibitor PD98059 weakly blocked IL-8 production (Fig. 5A). SB203580 and PD98059 treatment in HN12 cells, however, did not significantly block DFO-induced IL-8 production (Fig. 5C). Furthermore, IL-8 secretion appeared to correspond with the level of IL-8 mRNA accumulation in IHOK and HN12 cells (Fig. 5B and 5D).

### Both p38 and ERK1/2 activation contribute to iron chelator induced IL-8 production via a posttranscriptional mechanism

**Figure 6:**
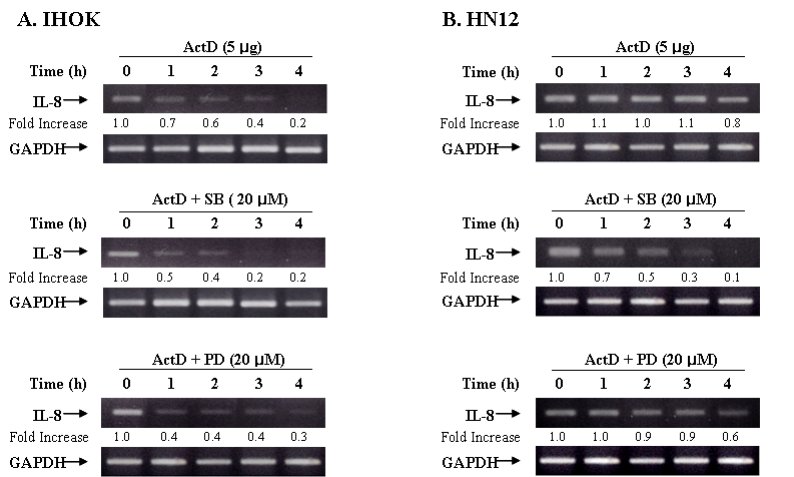
Iron chelator stabilized IL-8 mRNA through activation of p38 and ERK1/2. IHOK and HN12 cells were treated with DFO (1.0 mM) for 16 h to allow accumulation of IL-8 mRNA. Cells were then treated with the mRNA synthesis inhibitor actinomycin D (ActD; 5 μg/ml), and either ERK pathway inhibitor (PD98059) or p38 inhibitor (SB203580). GAPDH mRNA was used as an endogenous control message. Same procedure as described in the legend to Fig. 1 was performed. Results are representative of three independent experiments.

The accumulation of mRNA is the result of a balance between mRNA synthesis and degradation [37]. Given that our results demonstrated that NF-κB is involved in iron chelator-mediated IL-8 production in IHOK and HN12 cells (Fig. 3), we sought to determine whether MAP kinase activation by DFO is involved in the stabilization of IL-8 mRNA transcripts to augment IL-8 protein secretion. IHOK and HN12 cells were treated with DFO (1.0 mM) for 16 h, followed by treatment with actinomycin D (5 μg/ml), an inhibitor of mRNA synthesis, in the presence or absence of MAP kinase inhibitors (Fig. 6). Total RNA was isolated at various time points, and the remaining IL-8 mRNA was measured by semiquantitative RT-PCR. The inhibitory effect of SB203580 was stronger than that of PD98059 during actinomycin D treatment for 4 h (Fig. 6); however, the effect of MAP kinase inhibitors was weaker in HN12 cells than in IHOK cells. The inhibition of p38 and ERK resulted in the rapid disappearance of IL-8 mRNA compared to actinomycin D-treated control cells, suggesting that the posttranscriptional regulation of the IL-8 gene transcript is dependent on the iron chelator in IHOK and HN12 cells.

### The sensitivity of DFO dependent IL-8 mRNA induction to various inhibitors of intracellular signal transduction in IHOK and HN12 cells

**Figure 7:**
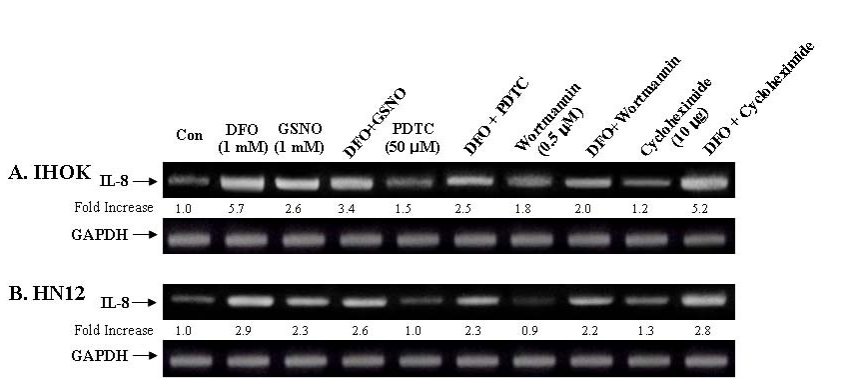
Effect of various inhibitors on the DFO-induced IL-8 mRNA expression. Cells were incubated for 16 h in the presence or absence of DFO 1.0 mM with and without GSNO (1.0 mM), PDTC (50 μM), hemoglobin (1 mg), wortmannin (0.5 μM), and cycloheximede (10 μg/ml). Same procedure as described in the legend to Fig. 1 was performed. The results are representative of three independent experiments.

We examined whether DFO induces IL-8 production through a variety of different signal transduction mechanisms (Fig. 7). Recent evidence indicates that nitric oxide (NO) influences Fe metabolism [38]. We therefore examined whether DFO induces IL-8 production via a NO-dependent mechanism. We initially observed that cells treated with 0.3 mM S-nitrosoglutathione (GSNO) expressed increased levels of IL-8 mRNA. We then observed that IHOK and HN12 cells incubated with 0.3 mM S-nitrosoglutathione (GSNO) in the presence of DFO showed decreased IL-8 mRNA production. These results indicated that the NO implicated in transcriptional activation of the DFO-induced IL-8 gene.

We also examined the involvement of the redox-sensitive NF-κB transcription factor in DFO-mediated up-regulation of IL-8 gene expression by co-incubation with DFO and pyrrolidine dithiocarbamate (PDTC), an antioxidant and potent inhibitor of NF-κB in IHOK cells. We observed that significant inhibition of the DFO-dependent IL-8 induction by PDTC occurred in IHOK cells (Fig. 7A and 7B). Similar results were obtained with wortmannin, an inhibitor of phosphatidylinositol 3-kinase-dependent activation of NAD(P)H oxidase. These findings suggest that NAD(P)H oxidase is the main source of ROS required for DFO-dependent transcriptional activation of the IL-8 gene. Finally, DFO had a negligible effect on IL-8 gene induction by cycloheximide (CHX) *de novo *protein synthesis in IHOK and HN12 cells.

## Discussion

Previous studies have demonstrated that IL-8 is secreted by normal and cancerous cells, both of which exhibited neutrophil chemotactic activity that corresponded with immunoreactive IL-8 concentrations, thereby indicating that carcinoma cells produce biologically active IL-8 [39,40]. Although the mechanisms governing tumor production remain unknown, IL-8 is suspected to play a role in the growth and production of carcinoma cells.

Iron and iron chelators have been shown to modulate inflammatory mediators and to regulate inflammatory process in several cell types [41,42]. Additionally, IL-8 and iron chelators have been implicated in the regulation of inflammation and other processes in the intestinal epithelium, monocytes, and human blood leukocytes [43-45]; however, no previous study had examined the differential modulatory effects of IL-8 in DFO signaling in immortalized and malignant oral keratinocytes. Here we demonstrated that an iron chelator used by bacteria to allow growth *in vitro *activates an inflammatory response even in the absence of conventional immunostimulatory or inflammatory stimuli in immortalized and oral cancer cells. We also demonstrated that the up-regulation of DFO-induced IL-8 expression was higher in IHOK cells than in HN12 cells (Fig. 1). Furthermore, DFO acted additively with IL-1β to up-regulate IL-8 in IHOK cells but not in oral cancer cells (Fig. 2). Thus, differences in the IL-8 response to DFO could be found between IHOK and HN4 cells. Although both of IHOK and HN12 cell are transformed human cells, the IHOKs relatively well expressed the normal phenotypes of oral keratinocytes, representing premalignant keratinocytes, while the HN4 cells disclosed typical malignant features of oral cancer cells [46]. In the previous our study, DFO-induced anticancer effect was prominent in IHOKs but relatively sparse in oral cancer cells [27,28]. The present data have the similar implications to IL-8 induction by iron-chelator, DFO, which is immunologically more responsible to IHOK than HN12 cells. These results emphasize the cell-type-specific nature of the signal transduction pathways that govern the response to DFO, and that the extent of iron chelator-responsiveness is also dependent on the state of cellular differentiation.

In the previous study we numerous *in vitro *and *in vivo *experiments that examined iron metabolism, focusing on the protective roles of heme oxygenase-1 [27,28,43,47,48], and determined that iron is a key and essential mineral in body cells. Several other studies have examined the role of iron chelators as anti-proliferative and anticancer agents by performing series of experiments using a single dose or limited dose of DFO (usually 0.1–0.2 mM) [49,50]. More specifically, in the iron-chelator-induced mitochondrial protection to combat ROS-mediated myocardial damage, a dose-dependent increase in cell survival was observed up to a DFO concentration of 10 mM [51]. Moreover, in our previous study [27,28], DFO had an IC_50 _(concentration required for 50% growth inhibition) of 0.99 mM for IHOK cells and 1.78 mM for HN12 cells. Since iron chelators play an anti-oxidative role promoting cell survival with H_2_O_2 _exposure (0.1 mM), cellular necrosis and apoptosis do not increase with a high concentration of DFO (up to 10 mM *in vitro*) [51]. Hippocampal neurons from E16 CD1 mice were also exposed to 10 mM DFO without cellular damage in an *in vitro *study of DFO protection against hypoxia-inducible factor (HIF)-1 alpha [52]. Our previous studies on DFO examining iron chelator-induced apoptosis showed a linear increase in apoptosis up to 1 mM, at which point an abrupt increase in cell necrosis occurred [27,28]. Because the present study examined IL-8 induction in IHOK and HN12 cells with DFO treatment, we did not report cell survival data related to apoptosis, but instead referred to our previous studies [27,28]. In the present study, to determine the maximum dose of DFO inducing IL-8 production, we applied DFO concentrations of up to 2 mM; we found that the maximum IL-8 production occurred with 0.75 mM DFO in IHOK cells, and then it decreased gradually as the concentration was increased to 2 mM. Therefore, although DFO is relatively safe when cells are exposed to concentrations as high as 2 mM, the optimal dose of DFO for inducing IL-8 in cultured cells is 0.75 mM or less. At greater concentrations, iron metabolism might be deleteriously affected, and an apoptosis-related reduction in cell survival could occur. As the mechanism of DFO in apoptosis or iron-metabolism cytotoxicity is a different issue from its role in IL-8 production, in the present study, we focused on the issue of IL-8 induction by DFO, which was maximal at 0.75 mM, and referred to our previous studies for information on DFO-induced apoptosis.

The binding of NF-κB to the IL-8 promoter is essential for the constitutive activation of IL-8 gene transcription, and p65 is one of the major components responsible for κB binding to the IL-8 promoter [53]. I-κBα, a member of the I-κB family, is phosphorylated in response to TNF or IL-1 stimulation, and is thereby subjected to ubiquitination and degradation by the 26S proteosome [54]. Degradation of I-κBα releases NF-κB for translocation to the nucleus and the subsequent activation of target genes [55]. In this study, we investigated DFO-induced IL-8 production in IHOK and HN12 cells by blocking the I-κB/NF-κB signal pathway. We noted I-κBα degradation after 16 h of DFO exposure, whereas treatment with IL-1β significantly induced I-κBα degradation after 1 h in IHOK and HN12 cells (Fig. 3). Moreover, phosphorylation of the I-κBα protein was very apparent after DFO treatment for 16 h in IHOK and HN12 cells (Fig. 3). Finally, we observed that DFO treatment led to an increase in DNA binding activity of NF-κB in IHOK and HN12 cells (Fig. 4). These findings indicate that DFO mediated I-κBα degradation and NF-κB binding induced the NF-κB-activating signal pathway to regulate the expression of the immunomodulatory target gene IL-8 in immortalized and malignant oral keratinocytes.

MAPK pathways are important in cancer pathogenesis because they control processes that are central to malignant progression such as cell growth, apoptosis, and cellular migration [33-36]. In this study, we observed that the inhibition of p38 or ERK with SB203580 or PD98059 significantly reduced levels IL-8 mRNA in IHOK cells (Fig. 5). We also found that an iron chelator could inhibit the stability of IL-8 mRNA, presumably through the activation of p38 and ERK1/2 pathways (Fig. 6). In addition, we found that differential DFO-induced regulation of p38 and ERK1/2 promoted the expression of IL-8 in IHOK and HN12 cells. We also observed that p38 and ERK1/2 are required for DFO-induced IL-8 secretion in IHOK and HN12 cells, thus providing evidence that these kinases act posttranscriptionally via a pathway involving the stabilization of the IL-8 transcript. Transcription factors, usually AP-1, C/EBPβ can be affected by p38 or ERK, and further study of post-transcriptional mechanisms for DFO-induced IL-8 involving p38 or ERK is thus warranted.

The mechanism that translates the iron chelator into a signal for IL-8 expression in oral cancer cells is not yet known. In this study, we showed that DFO-induced IL-8 was lowered by GSNO, suggesting that the NO was effective at entering cells, binding Fe, and causing Fe mobilization [38]. This also indicates that NO is the initial factor in the signaling cascade that mediates IL-8 gene transcriptional activation by DFO. NF-κB is also an important transcriptional factor for IL-8 production in cancer cells. PDTC, one of the most effective inhibitors of NF-κB, inhibits the NO-dependent induction of the IL-8 gene [56]. This is consistent with our finding that PDTC blocked the IL-8 induced by DFO, suggesting that activation of NF-κB regulates IL-8 expression in IHOK and oral cancer cells. The PI-3 kinase inhibitor wortmannin has been reported to inhibit the production of NO-induced IL-8 mRNA in monocytes [56]. We showed that wortmannin inhibited the production of DFO-induced IL-8 in IHOK and oral cancer cells, thus suggesting that an activation of PI-3 kinase is also involved in the IL-8 pathway.

## Conclusion

This study demonstrated that the chelation of intracellular iron induces NF-κB, p38, and ERK 1/2 MAPK activation, and results in an immunomodulatory IL-8 response in oral cancer cells. We therefore suggest that iron chelator-induced IL-8 may be an attractive immuno-target for oral premalignant lesion treatment.

## Abbreviations

IL-8, interleukin-8; DFO, deferoxamine; IHOK, immortalized human oral kertinocytes; ERKs, extracellular-regulated kinases; SCC, squamous cell carcinoma; MIM, mimosine; FC, ferric citrate; GSNO, S-nitrosoglutathione; PDTC, pyrrolidine dithiocarbamate (PDTC); CHX, cycloheximide

## Competing interests

The author(s) declare that they have no competing interests.

## Authors' contributions

HJL and JL co-designed the study with ECK, wrote the manuscripts, performed cell culture works, enzyme-linked immunoabsorbent assay and RT-PCR. SKL in WK Univ. performed EMSA, stastistical analysis and assisted in figure preparation. ECK coordinated the study with SKL supervised the study and edited the manuscripts. All the authors read and approved the final manuscript.

## Pre-publication history

The pre-publication history for this paper can be accessed here:


